# Cocoa butter-like lipid production ability of non-oleaginous and oleaginous yeasts under nitrogen-limited culture conditions

**DOI:** 10.1007/s00253-017-8126-7

**Published:** 2017-02-06

**Authors:** Yongjun Wei, Verena Siewers, Jens Nielsen

**Affiliations:** 1 Department of Biology and Biological Engineering, Chalmers University of Technology, SE-412 96 Gothenburg, Sweden; 2 Novo Nordisk Foundation Center for Biosustainability, Chalmers University of Technology, SE-412 96 Gothenburg, Sweden; 3 Novo Nordisk Foundation Center for Biosustainability, Technical University of Denmark, 2800 Kgs. Lyngby, Denmark

**Keywords:** Cocoa butter-like lipids, Oleaginous yeasts, Lipid production, TAG profiles

## Abstract

Cocoa butter (CB) extracted from cocoa beans is the main raw material for chocolate production. However, growing chocolate demands and limited CB production has resulted in a shortage of CB supply. CB is mainly composed of three different kinds of triacylglycerols (TAGs), POP (C16:0–C18:1–C16:0), POS (C16:0–C18:1–C18:0), and SOS (C18:0–C18:1–C18:0). The storage lipids of yeasts, mainly TAGs, also contain relative high-level of C16 and C18 fatty acids and might be used as CB-like lipids (CBL). In this study, we cultivated six different yeasts, including one non-oleaginous yeast strain, *Saccharomyces cerevisiae* CEN.PK113-7D, and five oleaginous yeast strains, *Trichosporon oleaginosus* DSM11815, *Rhodotorula graminis* DSM 27356, *Lipomyces starkeyi* DSM 70296, *Rhodosporidium toruloides* DSM 70398, and *Yarrowia lipolytica* CBS 6124, in nitrogen-limited medium and compared their CBL production ability. Under the same growth conditions, we found that TAGs were the main lipids in all six yeasts and that *T. oleaginosus* can produce more TAGs than the other five yeasts. Less than 3% of the total TAGs were identified as potential SOS in the six yeasts. However, *T. oleaginosus* produced 27.8% potential POP and POS at levels of 378 mg TAGs/g dry cell weight, hinting that this yeast may have potential as a CBL production host after further metabolic engineering in future.

**Electronic supplementary material:**

The online version of this article (doi:10.1007/s00253-017-8126-7) contains supplementary material, which is available to authorized users.

## Introduction

Cocoa butter (CB) is extracted from cocoa beans of the cocoa tree *Theobroma cacao*, and besides being used as food flavor and cosmetics additive, CB is the main component of chocolate (Lipp and Anklam 1998). Due to variations in CB production and an increasing chocolate demand by consumers, the CB supply is currently insufficient and its price has increased in recent years (Clough et al. 2009). Although some other vegetable oils can be used as CB equivalents, for example, Illipe butter, shea butter, and kokum butter, their production is also limited (Jahurul et al. 2013; Verstringe et al. 2012). Therefore, there are interests in developing other routes for production of CB-like lipids (CBL), which can be used as a stable and economically feasible supply for chocolate production (Clough et al. 2009).

CB mainly contains three different kinds of triacylglycerols (TAGs), which are esters formed with one glycerol and three fatty acids (Jahurul et al. 2013). The fatty acids in the *sn*-1 and *sn*-3 positions of CB TAG backbone glycerol are mainly palmitic acid (C16:0) or stearic acid (C18:0), and the fatty acid in *sn*-2 position is predominantly oleic acid (C18:1). POP (C16:0–C18:1–C16:0), POS (C16:0–C18:1–C18:0), and SOS (C18:0–C18:1–C18:0) are the three main CB TAGs, and they are therefore also the desired TAGs of CBL (Jahurul et al. 2013). POP, POS, and SOS ratios in CB are 14–16.4, 34.6–38.3, and 23.7–28.4%, respectively (Lipp and Anklam 1998). Moreover, the fatty acid distributions of CB are C16:0 (24.1–25.8%), C18:0 (33.3–37.6%), and C18:1 (32.7–36.5%) (Lipp and Anklam 1998).

Currently, enzymatic re-esterification of vegetable oils using lipases is used for CBL production (Ferreira-Dias et al. 2013; Matsuo et al. 1981; Mohamed 2013; Verstringe et al. 2012; Xu 2000). However, re-esterification requires hydrogenation of large amounts of plant oils which have limited production (Ferreira-Dias et al. 2013; Wang et al. 2006). In addition to the lipase-assisted method, yeasts, especially oleaginous yeasts, also have potential application for CBL production, as the main fatty acids produced by yeasts are C16 and C18 fatty acids (Beopoulos et al. 2011; Papanikolaou and Aggelis 2011). For the model yeast, *Saccharomyces cerevisiae*, global analysis of its lipidome, has shown that only small amounts of CBL were produced (Ejsing et al. 2009). On the other hand, several different oleaginous yeast strains have been reported as candidates for CBL production, such as *Yarrowia lipolytica*, *Rhodosporidium toruloides*, *Lipomyces starkeyi*, and *Cryptococcus curvatus* (Hassan et al. 1994; Hassan et al. 1995; Papanikolaou et al. 2001; Papanikolaou et al. 2003; Wu et al. 2011). Though the cultivation conditions that affect fatty acid production have been examined in several yeasts (Kolouchová et al. 2016), a detailed comparison of the CBL production ability in different yeasts and given conditions has rarely been reported.

In this study, we compared the compositions of total lipids, fatty acids, and TAGs of one non-oleaginous *S. cerevisiae* strain and five oleaginous yeast strains, which had been reported to accumulate lipids up to more than 20% of their cell dry weight, under nitrogen-limited growth conditions (Ageitos et al. 2011; Galafassi et al. 2012; Nijkamp et al. 2012). Based on the analysis, we evaluated CBL production of these six yeast species, which represent a good starting point to be further engineered for CBL production in future.

## Materials and methods

### Yeast strains and cultivation media

One non-oleaginous *S. cerevisiae* strain and five oleaginous yeast strains were used in this study. *S. cerevisiae* CEN.PK113-7D was kindly provided by Peter Kötter (Entian and Kötter 2007). *Trichosporon oleaginosus* DSM11815, *Rhodotorula graminis* DSM 27356, *L*. *starkeyi* DSM 70296, and *R. toruloides* DSM 70398 were purchased from the culture collection of the DSMZ (Braunschweig, Germany). *Y*. *lipolytica* CBS 6124 was purchased from the CBS-KNAW Fungal Biodiversity Centre (Utrecht, The Netherlands). Rich media, YPD or YM, were used for cultivation of these six yeasts. The YPD medium was prepared with 10 g/l yeast extract (Merck Millipore), 20 g/l peptone (Merck Millipore), and 20 g/l glucose (Merck Millipore). The YM medium was constituted of 3 g/l yeast extract (Merck Millipore), 3 g/l malt extract (Oxoid), 5 g/l peptone from soybeans (Merck Millipore), and 10 g/l glucose (Merck Millipore). The nitrogen-limited medium (named NLM medium in the text) was prepared as described in the literature using 70 g/l glucose (Merck Millipore) as carbon source (Yang et al. 2014).

### Bioscreen cultivation of yeasts

Yeasts were streaked on YPD plates from frozen stocks. A single colony of each strain was used to inoculate 50 ml falcon tubes containing 5 ml YPD medium and cultivated at 200 rpm and 30 °C. Overnight cultures (for *L. starkeyi*, a 36-h cultivation was used) were used to inoculate fresh YPD, YM, or NLM medium with an optical density (OD_600 nm_) of about 0.1. Each strain in each medium was analyzed in eight replicates and cultivated in a Bioscreen C MBR (Oy Growth Curves Ab Ltd.). The parameters of the Bioscreen C MBR cultivation were as follows: a working volume of 150 μl, a cultivation temperature of 30 ± 0.1 °C, and OD reading period = 12 min. Other cultivation conditions and data processing methods were the same as described previously (Adeboye et al. 2014; Warringer and Blomberg 2003). The cultivations in Bioscreen microplates were finished when the yeasts reached their stationary phase, and the running time for all strains in this study was 39.4 h.

### Shake flask growth characteristics measurement

Due to hyphal growth of *Y. lipolytica* and *R. graminis* in YPD and YM media, maximum growth rates of *Y. lipolytica* and *R. graminis* in YPD and YM media were determined using a dry weight method. Overnight cultures of *Y. lipolytica* and *R. graminis* were used to inoculate 500-ml unbaffled shake flasks with 120-ml YPD or YM medium at about 0.2 g dry weight per liter medium in three biological duplicates. Five-milliliter culture of *Y. lipolytica* and *R. graminis* was collected every few hours using 0.45-μm polyethersulfone filters (Sartorius Stedim Biotech). The filters were dried in a microwave (Severin) for 15 min (126 W) and put into one desiccator (Duran) for 2 days until a stable weight was achieved.

In order to verify the growth results obtained using the Bioscreen C MBR and to determine the cultivation time for yeast biomass collection in unbaffled shake flasks, yeast growth characteristics in 100-ml shake flask with 20 ml NLM medium were determined in biological triplicates.

### Microscopic analysis of yeast cells

Overnight cultures of the six yeasts (for *L. starkeyi*, a 36-h cultivation was used) were used to inoculate 100-ml unbaffled shake flasks with 20 ml YPD, YM, or NLM media at an OD_600 nm_ of about 0.1 in biological duplicates. After 72-h cultivation, 100 μl yeast cultures from each shake flask were sampled by centrifugation at 6500×*g* for 5 min. The yeast cells were washed twice with 500 μl PBS buffer and suspended in 100 μl PBS buffer. Then, 0.5 μl Nile red (1 mg/ml in DMSO) was added to the suspended cells and stained in the dark for 30 min. After that, the stained cells were washed twice with 500 μl PBS buffer and resuspended in 50 μl PBS buffer for microscopic imaging (Greenspan et al. 1985; Wu et al. 2010). The fluorescence microscopy images were acquired with a Leica AF 6000 inverted microscope (Leica Microsystems, and the excitation/emission is 546/605 nm) and processed with the Leica Application Suite (LAS) software.

### Analysis of lipid and fatty acid composition

Each strain was used to inoculate 50-ml falcon tubes with 10 ml YPD medium and cultivated at 30 °C with shaking at 200 rpm for 18 h (for *L. starkeyi*, a 36-h cultivation was used). The cultures were transferred to 250-ml shake flasks with 50 ml fresh YPD medium and cultivated for another 12 h at 30 °C with shaking at 200 rpm. Then, the cultures were collected by centrifugation and resuspended in 50 ml fresh NLM medium. Respective volumes were used to inoculate 5-l shake flask with 1 l fresh NLM medium to ensure a starting OD_600 nm_ of 0.1. The yeast cells were harvested by centrifuging at 10,000×*g* for 15 min after 120 h of cultivation. Thirty milliliters of the cultures was collected separately by centrifugation at 10,000×*g* for 15 min and dried using a freeze dryer (Alpha 2-4 LSC, Christ GmbH); the remaining yeast cells were used for lipid extraction for TAG analysis. The collected yeast cells were washed once with distilled water before further treatment. Freeze-dried yeast cells from the 30 ml samples were used to determine the total lipid profile and the total fatty acid composition using microwave-assisted methods (Khoomrung et al. 2012; Khoomrung et al. 2013).

### Lipid extraction for TAG analysis

The total dry cell weight (DCW) of the 1 l cultures was estimated based on the dried biomass from the 30 ml samples. For every gram of the yeast pellets (DCW), 6 ml HCl solution (4 M) was added. The samples were kept at 78 °C in a water bath for 2 h to disrupt the yeast cells. After cooling the samples to room temperature, 12 ml chloroform–methanol 1:1 (*v*/*v*) solution per gram of yeast biomass was added and the samples incubated for 10 min at 1500 rpm using a DVX-2500 multi-tube vortexer (VWR). After centrifugation at 6500×*g* for 10 min, the lower phase (chloroform phase) was collected into a new 50-ml falcon tube. In order to extract all the lipids in the yeast samples, another equal volume of chloroform was added to the supernatant (including water phase and the remaining yeast biomass), mixed using the DVX-2500 multi-tube vortexer and incubated for 10 min at 1500 rpm. After centrifugation at 6500×*g* for 10 min, the lower phase was collected and combined with the previously obtained one. Finally, an equal volume of 0.1% NaCl was added to the combined lower phase (chloroform phase), vortexed, and centrifuged at 6500×*g* for 10 min to collect the lower phase containing the lipids. The collected liquid was dried in glass tubes with a miVac concentrator (Genevac) at 50 °C until the weight of each sample did not change (Nambou et al. 2014; Yu et al. 2015).

Approximately 75 μg lipid samples were collected in a glass vial and melted at 80 °C. Then, they were immediately dissolved in 1.2 ml acetone–tetrahydrofuran (THF) 1:1 (*v*/*v*) (Rathburn Chemicals). Afterwards, they were filtered through a 0.45-μm filter (PTFE, Cameo) directly into a sequence vial. The TAG composition analysis was performed by ultra performance liquid chromatography (UPLC) using refractive index (RI) detection (Waters), and TAG compositions were expressed in relative area percentages (Shukla et al. 1983). In contrast to the previously published method (Shukla et al. 1983), acetonitrile (Merck)/methyl tert-butyl ether (MTBE) (Rathburn Chemicals) was used as mobile phase instead of acetonitrile/THF and an RI detector was used instead of a UV detector. Cocoa butter standards and TAG composition analyses were completed by AAK, and TAG standards were purchased from Larodan.

## Results

### Growth characteristics of the yeasts in different media

Yeasts are able to grow and produce different levels of lipids in different conditions and media (Barth and Gaillardin 1997). Especially, they have been reported to increase their lipid production when nitrogen is exhausted and excess carbon source is present in the medium (Mullner and Daum 2004; Ratledge and Wynn 2002). In this study, two rich media, YPD and YM, and one nitrogen-limited and carbon-excess medium, NLM medium, were employed to assess the growth of the six yeasts. All yeast strains were able to grow in these three media, but they showed different growth characteristics and maximum specific growth rates (Table 1 and Fig. S1a–f). In general, maximum growth rates of *T. oleaginosus* and *R. toruloides* were higher than those of the other yeasts in NLM medium.

**Table 1:** Maximum specific growth rates of six different yeasts in different media

	μ_max_^a^ (h^−1^, YPD medium)	μ_max_^a^ (h^−1^, YM medium)	μ_max_^a^ (h^−1^, NLM medium)	μ_max_^b^ (h^−1^, NLM medium)
*Saccharomyces cerevisiae*	0.53 ± 0.02	0.50 ± 0.01	0.37 ± 0.02	0.24 ± 0.01
*Trichosporon oleaginosus*	0.47 ± 0.01	0.42 ± 0.01	0.40 ± 0.02	0.39 ± 0
*Rhodotorula graminis*	0.19 ± 0.01^c^	0.2 ± 0^c^	0.21 ± 0.01	0.27 ± 0.01
*Lipomyces starkeyi*	0.13 ± 0.01	0.11 ± 0	0.13 ± 0.01	0.12 ± 0
*Rhodosporidium toruloides*	0.42 ± 0.03	0.38 ± 0.01	0.29 ± 0.03	0.45 ± 0.02
*Yarrowia lipolytica*	0.24 ± 0.01^c^	0.21 ± 0.01^c^	0.23 ± 0.02	0.31 ± 0.04

^a^Maximum specific growth rates were calculated based on calculated OD_600 nm_ results collected from Bioscreen cultivation, *n* = 8

^b^Maximum specific growth rates were calculated based on OD_600 nm_ data collected from shake flask cultivation, *n* = 3

^c^Maximum specific growth rates were calculated based on dry cell weight collected from shake flask cultivation, *n* = 3

Most yeasts cultivated in the three media displayed similar morphologies after 72 h, but some *Y. lipolytica* and *R. graminis* cells in YPD and YM media showed hyphal growth after 72 h of cultivation (Fig. S2). Nile red was used to stain lipid droplets present in the cells (Greenspan et al. 1985; Kimura et al. 2004), and the lipid droplets of all yeasts formed under NLM medium cultivation exhibited a brighter fluorescence and were larger than under rich medium (YPD or YM) cultivation (Fig. S2).

### Lipid profiles of the yeasts

The total lipid profiles in yeasts cover TAGs, steryl esters (SE), ergosterol (ES), cardiolipin (CL), phosphatidic acid (PA), phosphatidylethanolamine (PE), phosphatidylinositol (PI), phosphatidylserine (PS), and phosphatidylcholine (PC) (Czabany et al. 2007; de Kroon et al. 2013; Kaneko et al. 1976). The total lipid content and distribution of the six yeasts cultivated in NLM medium for 120 h was measured, and *S. cerevisiae*, *T. oleaginous*, *R. graminis*, *L. starkeyi*, *R. toruloides*, and *Y. lipolytica* produced 76, 428, 213, 183, 166, and 134 mg lipids/g DCW, respectively (Table S1). TAG was the most abundant lipid class in all yeasts. *S. cerevisiae* produced 38.7 mg TAGs/g DCW, while the five oleaginous yeasts produced TAG levels between 106 and 378.6 mg lipids/g DCW (Table S1). The TAG proportion of the total lipids in the five oleaginous yeasts was between 79.0 and 90.3%, while the TAG ratio in *S. cerevisiae* was only 50.7%. *S. cerevisiae* contained 34.0% SE and 6.4% ES, which were higher than the SE and ES proportion of the five oleaginous yeasts (Table 2). Considering the remaining lipid classes, CL, PA, PE, PI, PS, and PC, their contents were very low in all six yeasts (Table 2).

**Table 2:** The relative lipid content of six yeasts cultivated in NLM medium (*n* = 2)

	Relative lipid content (%, wt/wt)^a^							
	Steryl esters	Triacylglycerol	Ergosterol	Cardiolipin	PE	PC	PS	PI
*Saccharomyces cerevisiae*	34.0 ± 2.5	50.7 ± 1.0	6.4 ± 0	ND	2.0 ± 0.4	5.8 ± 0.7	1.1 ± 0.4	ND
*Trichosporon oleaginosus*	ND	88.1 ± 1.7	3.8 ± 0.6	0.1 ± 0	1.1 ± 0.1	3.5 ± 0.3	3.3 ± 0.4	0.2 ± 0.2
*Rhodotorula graminis*	7.0 ± 4.0	86.1 ± 4.3	1.2 ± 0.3	ND	1.7 ± 0	4.0 ± 0.1	ND	ND
*Lipomyces starkeyi*	0.4 ± 0.1	90.3 ± 0.2	0.8 ± 0.1	ND	2.2 ± 0.1	3.5 ± 0.2	1.8 ± 0.2	1.1 ± 0.2
*Rhodosporidium toruloides*	1.3 ± 0.4	89.8 ± 2.1	1.2 ± 04	ND	1.5 ± 0.5	4.7 ± 0.7	0.6 ± 0	0.7 ± 0.5
*Yarrowia lipolytica*	4.1 ± 0.3	79.0 ± 2.1	2.9 ± 0.5	1.3 ± 0.3	ND	12.6 ± 1.0	ND	ND

*PE* phosphatidylethanolamine, *PC* phosphatidylcholine, *PS* phosphatidylserine, *PI* phosphatidylinositol, *ND* not determined (the peak was not detected or the peak area was too small to determine)

^a^Phosphatidic acid (PA) and free fatty acid levels were not determined in this study (below the detection limit or no detectable peaks)

### Total fatty acid profiles of the yeasts

Though the oleaginous yeasts produced more total fatty acids than *S. cerevisiae*, the most abundant fatty acids of all the six yeasts were C16 and C18 fatty acids, which accounted for >95% of the total fatty acids (Table 3 and Table S2). The C16/C18 ratio of *S. cerevisiae* and *Y. lipolytica* was 1.9 and 0.6, respectively, which was higher than the C16/C18 ratio of the other four yeasts (Table 3). For *S. cerevisiae*, the three main fatty acids were C16:0 (13.7%), palmitoleic acid (C16:1, 50.0%), and C18:1 (30.0%); for *Y. lipolytica*, the four main fatty acids were C16:0 (13.6%), C16:1 (24.1%), C18:1 (46.2%), and linoleic acid (C18:2, 11.2%); and for the other four oleaginous yeasts, C18:1 was the most abundant fatty acid, followed by C16:0, and the combined relative C16:0 and C18:1 content of these four oleaginous yeasts was 64.3–79.5% (Table 3). The C16:0/C16:1 ratios of *S. cerevisiae* and *Y. lipolytica* were 0.3 and 0.6, respectively, while the ratios of the other four yeasts were 8.3 to 53.2, showing much higher proportion of the mono-unsaturated C16:1 fatty acid in *S. cerevisiae* and *Y. lipolytica* than in the other four yeasts (Table 3).

**Table 3:** The relative total fatty acid contents of six yeasts cultivated in NLM medium (*n* = 2)

	Relative content of fatty acids (%, wt/wt)						
	C16:0	C16:1	C18:0	C18:1	C18:2	C18:3	Others^a^
*Saccharomyces cerevisiae*	13.7 ± 0.1	50.0 ± 0.3	3.5 ± 0.3	30.0 ± 0.5	ND	ND	2.6 ± 0.5
*Trichosporon oleaginosus*	29.7 ± 1.1	0.8 ± 0.1	6.4 ± 0.1	48.3 ± 0	9.8 ± 0.8	0.3 ± 0	4.6 ± 0.7
*Rhodotorula graminis*	22.8 ± 0.2	2.7 ± 0.7	1.8 ± 0	46.9 ± 0.3	18.8 ± 1.0	2.0 ± 0	5.1 ± 0.4
*Lipomyces starkeyi*	31.6 ± 0.2	1.6 ± 0	10.1 ± 0	47.8 ± 0.3	4.2 ± 0.8	0.1 ± 0	4.4 ± 0.4
*Rhodosporidium toruloides*	27.7 ± 0.9	0.5 ± 0	14.6 ± 1.2	36.6 ± 1.7	14.7 ± 1.4	1.8 ± 0.1	4.0 ± 0.2
*Yarrowia lipolytica*	13.6 ± 0.1	24.1 ± 0.6	2.1 ± 0.2	46.2 ± 0.5	11.2 ± 0.8	ND	2.7 ± 0.2

*ND* not determined (the peak was not detected or the peak area was too small to determine)

^a^Others represent fatty acids of C12:0, C14:0, C14:1, C20:0, C20:1, C22:0, C24:0, and C26:0

In general, the proportion of unsaturated fatty acids in all six strains was higher than the proportion of saturated fatty acid. Especially, *S. cerevisiae*, *R. graminis*, and *Y. lipolytica* contained more than 72% unsaturated fatty acids, while *T. oleaginosus*, *L. starkeyi*, and *R. toruloides* produced 60.8, 55.3, and 54.9% unsaturated fatty acids, respectively (Table 3 and Table S2). *S. cerevisiae* did not produce polyunsaturated fatty acids, but the other five oleaginous yeasts produced some polyunsaturated fatty acids, for example, *T. oleaginosus*, *R. graminis*, *R. toruloides*, and *Y. lipolytica* can produce 9.8, 18.8, 14.7, and 11.2% C18:2 of their total fatty acids, respectively (Table 3).

### TAG profiles of the yeasts

**Fig. 1:**
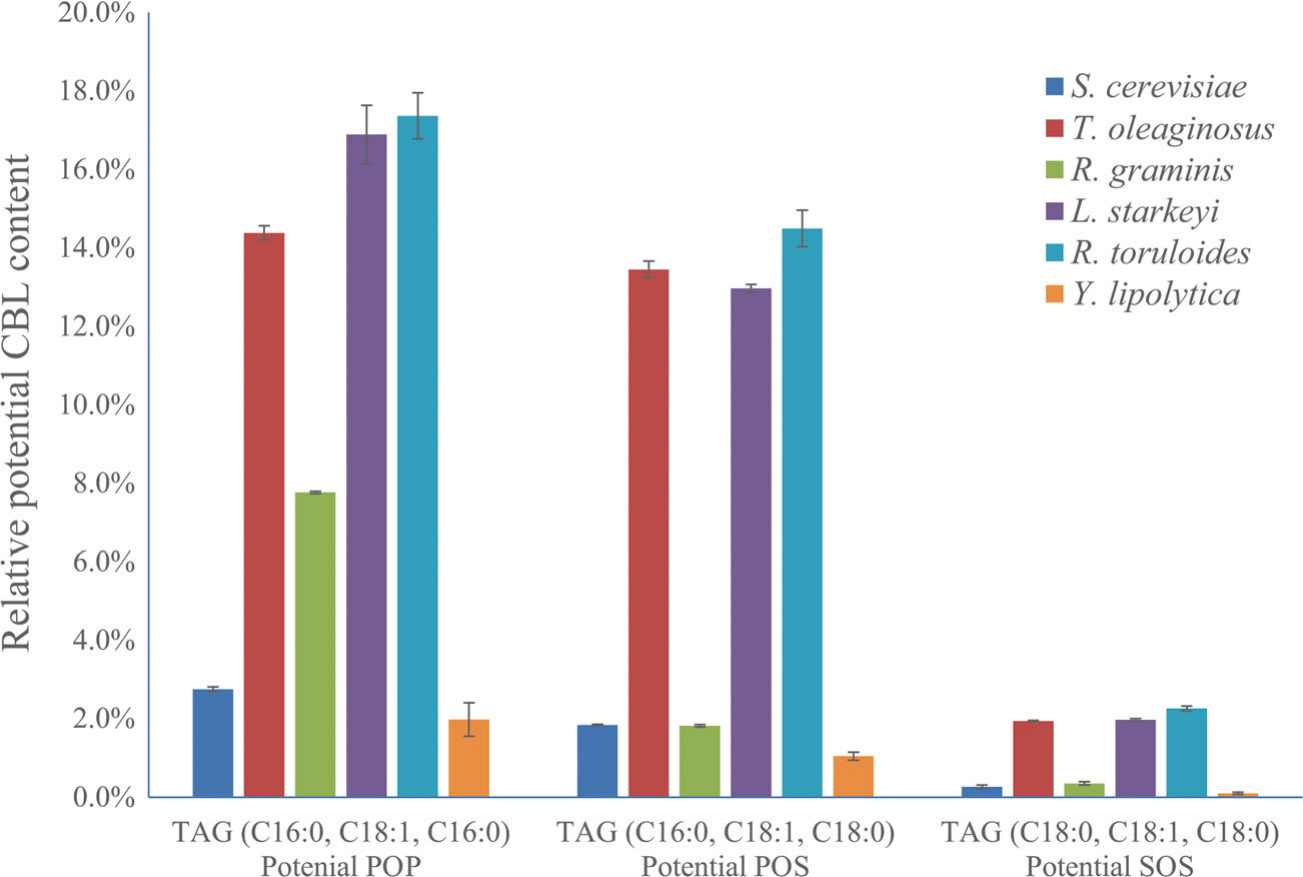
Relative content of potential *POP*, *POS*, and *SOS* of six yeasts

The TAG profile data of this study only provide the fatty acid composition of each TAG, for example, TAG (C16:0, C18:1, C16:0) contains two molecules of C16:0 and one molecule of C18:1, but their respective positions in the glycerol backbone are not determined. *S. cerevisiae*, *T. oleaginous*, *R. graminis*, *L. starkeyi*, *R. toruloides*, and *Y. lipolytica* can produce at least 27, 35, 36, 36, 36, and 31 different kinds of TAGs, respectively (Fig. 1, Table 4 and Table S3). However, most TAGs comprise less than 5% of the total TAGs in a strain (Table S3). The main TAGs of the six yeast strains are listed in Table 4. The main TAGs of *S. cerevisiae* were TAG (C18:1, C16:1, C16:1; 24.3%) and TAG (C18:0, C16:1, C16:1; 18.9%). The main TAGs of *R. toruloides* were TAG (C16:0, C18:1, C18:1; 22.2%), TAG (C18:1, C18:1, C18:1; 12.0%), and TAG (C16:0, C18:2, C18:1; 10.7%). The main TAGs of *Y. lipolytica* were TAG (C16:0, C18:1, C16:1; 18.4%), TAG (C18:1, C18:1, C18:1; 11.8%), and TAG (C16:0, C18:1, C18:1; 11.4%). Interestingly, the main TAGs of *T. oleaginous*, *L. starkeyi*, and *R. toruloides* were the same, TAG (C16:0, C18:1, C18:1), TAG (C16:0, C18:1, C16:0, potential POP), and TAG (C16:0, C18:1, C18:0, potential POS); the combined relative content of these three TAGs in *T. oleaginous*, *L. starkeyi*, and *R. toruloides* were 56.1, 59.5, and 47.6%, respectively (Table 4).

**Table 4:** The relative TAG content (>5%) of six yeasts cultivated in NLM medium (*n* = 2)

	Relative TAG content of total TAGs (%)					
	*Saccharomyces cerevisiae*	*Trichosporon oleaginosus*	*Rhodotorula graminis*	*Lipomyces starkeyi*	*Rhodosporidium toruloides*	*Yarrowia lipolytica*
TAG (C16:0, C18:1, C18:1)	7.0 ± 0.2	28.3 ± 0.1	22.2 ± 0.3	29.7 ± 0.2	15.7 ± 0.4	11.4 ± 1.4
TAG (C16:0, C18:1, C16:0)	2.7 ± 0.1	14.4 ± 0.2	7.8 ± 0	16.9 ± 0.7	17.4 ± 0.6	2.0 ± 0.4
TAG (C16:0, C18:1, C18:0)	1.8 ± 0	13.4 ± 0.2	1.8 ± 0	13.0 ± 0.1	14.5 ± 0.5	1.0 ± 0.1
TAG (C18:1, C18:1, C18:1)	1.8 ± 0.2	8.7 ± 0.3	12.0 ± 0	11.3 ± 0.1	3.4 ± 0.1	11.8 ± 0.1
TAG (C18:0, C18:1, C18:1)	1.3 ± 0.1	7.4 ± 0.1	2.1 ± 0.2	6.9 ± 0.2	4.5 ± 0.1	2.6 ± 0.3
TAG (C16:0, C18:2, C18:1)	0.0	6.3 ± 0.1	10.7 ± 0.1	2.9 ± 0.3	8.1 ± 0.2	0
TAG (C18:2, C18:1, C18:1)	0.2 ± 0.2	3.4 ± 0	8.3 ± 0.2	1.4 ± 0.3	2.0 ± 0.1	0
TAG (C14:0, C18:1, C18:1)	0	1.4 ± 0.1	4.4 ± 0	2.5 ± 0.1	1.7 ± 0	5.0 ± 0
TAG (C16:0, C18:2, C16:0)	0.8 ± 0.1	1.4 ± 0.1	3.1 ± 0	1.1 ± 0.1	6.2 ± 0.1	1.7 ± 0.3
TAG (C16:0, C18:2, C18:0)	0.2 ± 0	1.3 ± 0.2	0.9 ± 0	1.2 ± 0	5.0 ± 0.1	0.6 ± 0
TAG (C16:0, C18:2, C18:2)	13.1 ± 0.5	0.9 ± 0	1.6 ± 0	0.2 ± 0.1	1.3 ± 0.2	6.2 ± 0.9
TAG (C16:1, C18:1, C18:1)	0.9 ± 0.4	0.3 ± 0	0.6 ± 0	0.1 ± 0	0.1 ± 0.1	5.6 ± 0.2
TAG (C16:1, C16:1, C16:1)	6.6 ± 0.3	0.2 ± 0	1.2 ± 0	0.2 ± 0	1.1 ± 0.1	1.2 ± 0
TAG (C18:1, C16:1, C16:1)	24.3 ± 1.7	0	0.3 ± 0	0	0.1 ± 0	9.6 ± 0.1
TAG (C18:0, C16:1, C16:1)	18.9 ± 1.5	0	0 ± 0	0	0	9.5 ± 0.1
TAG (C16:0, C18:1, C16:1)	6.1 ± 0.5	0	0.5 ± 0	0.2 ± 0	0.3 ± 0.4	18.4 ± 0.7
Unknown TAGs	8.6 ± 1.8	0.7 ± 0.1	3.1 ± 0	0.9 ± 0	1.0 ± 0.3	4.6 ± 0.5

The potential CBL (mainly POP, POS, and SOS) content differed in the six yeasts. The proportion of potential POP and POS in *S. cerevisiae*, *R. graminis*, and *Y. lipolytica* was only 4.6, 9.6, and 3.0%, respectively, whereas it added up to 27.8, 29.8, and 31.9% in *T. oleaginous*, *L. starkeyi*, and *R. toruloides*, respectively. However, the relative content of TAG (C18:0, C18:1, C18:0, potential SOS) in all yeasts remained low, between 0.1 and 2.3% (Fig. 1).

### Fatty acid composition of the TAGs in different yeasts

Analyses showed that more than 90.9% of the fatty acids of the TAGs in all yeasts were C16 and C18 fatty acids (Table 5). Besides, the C16/C18 ratios of TAGs were different from the corresponding ratio among the total fatty acids. Thus, the C16/C18 ratios of TAGs were lower than in the total fatty acid pool in *S. cerevisiae*, *T. oleaginosus*, and *L. starkeyi*, but the C16/C18 ratios of TAGs were higher than in the total fatty acids in *R. toruloides*, *R. graminis*, and *Y. lipolytica*. In general, the relative unsaturated fatty acid content of TAGs were nearly the same as in the total fatty acid pools in *T. oleaginosus*, *L. starkeyi*, and *R. toruloides*, but the relative unsaturated fatty acid content of TAGs were higher than in the total fatty acid pools in *S. cerevisiae*, *R. graminis*, and *R. lipolytica* (Tables 3 and 5).

**Table 5:** The relative fatty acid contents of TAGsof six yeasts cultivated in NLM medium (*n* = 2)

	Relative fatty acid content of TAGs (%)							
	C16:0	C16:1	C18:0	C18:1	C18:2	C18:3	Others^a^	Unknown^b^
*Saccharomyces cerevisiae*	13.4 ± 0.8	39.4 ± 2.3	7.7 ± 0.3	20.9 ± 0.3	9.4 ± 0.5	0	0.5 ± 0	8.6 ± 1.8
*Trichosporon oleaginosus*	28.7 ± 0.6	0.4 ± 0	10.4 ± 0	50.7 ± 0.4	5.2 ± 0.2	0.8 ± 0	3.0 ± 0	0.7 ± 0.1
*Rhodotorula graminis*	24.7 ± 0	2.2 ± 0	4.3 ± 0.1	48.0 ± 0.1	7.9 ± 0.1	4.0 ± 0.1	5.5 ± 0.1	3.1 ± 0
*Lipomyces starkeyi*	29.1 ± 0.6	0.3 ± 0	9.8 ± 0.2	53.0 ± 0.4	3.3 ± 0.1	0.3 ± 0.1	3.3 ± 0	0.9 ± 0.2
*Rhodosporidium toruloides*	35.1 ± 0.4	2.4 ± 0	11.5 ± 0.1	37.4 ± 0.7	8.9 ± 0.7	1.6 ± 0	2.2 ± 0	1.0 ± 0.3
*Yarrowia lipolytica*	16.8 ± 0.3	23.5 ± 0.1	5.3 ± 0	41.1 ± 1.2	5.7 ± 0.7	0.4 ± 0.1	2.6 ± 0.2	4.6 ± 0.5

^a^Others represent fatty acids of C14:0, C20:0, C22:0, and C24:0

^b^Unknown represents TAGs whose fatty acid composition was not determined

In the five oleaginous yeasts, C18:1 was the most abundant fatty acid and the relative C18:1 content of the TAGs was 37.4–53.0%, which was consistent with the total fatty acid composition where the relative C18:1 content of the five oleaginous yeasts was 36.6–48.3%. In contrast, while C16:1 was the most abundant fatty acid of TAGs in *S. cerevisiae* with a relative content of 39.4%, the relative C16:1 content of total fatty acid in *S. cerevisiae* was 50% (Tables 3 and 5). C16:0 was the 2nd most abundant fatty acid of TAGs in *T. oleaginous*, *R. graminis*, *L. starkeyi*, and *R. toruloides*, and the relative content was 24.9–35.1%, which was similar to the C16:0 content of total fatty acid in the four yeasts (22.8–31.6%). For *Y. lipolytica*, the two main fatty acids of the TAGs other than C18:1 were C16:1 (23.5%) and C16:0 (16.8%), similar to the relative C16:1 (24.1%) and C16:0 (13.6%) content in the total fatty acid pool (Tables 3 and 5).

## Discussion

Here, we investigated lipid production and composition of six different yeast strains cultivated in the same nitrogen-limited medium and analyzed their ability to produce CBL. Importantly, we showed that *T. oleaginosus* can produce 378 mg TAGs/g DCW, and its relative content of potential POP and POS was 14.4 and 13.4%, respectively, suggesting that it has the highest potential as a CBL producer among these six yeasts. Besides, our results gave insights into lipid production of the yeasts, suggesting potential metabolic engineering directions for future CBL production in yeasts.

We analyzed the growth characteristics of the yeasts, and the results showed that most strains can grow faster in rich medium (YPD and YM media) than in nitrogen-limited medium (NLM medium) (Fig. S1 and Table 1). However, when comparing lipid droplets, the cells grown in NLM medium produced bigger but fewer lipid droplets than the ones grown in YPD or YM media, suggesting that a nitrogen-limited environment can alter the morphology of yeast lipid droplets and is better suited for lipid production as reported earlier (Fig. 1 and Fig. S2) (Papanikolaou and Aggelis 2011). The growth rates of *L. starkeyi* in all the three media were low, and it had the lowest growth rates among the six yeast strains in all three media, hinting that the growth rates of *L. starkeyi* should be improved before using it as a lipid or TAG producer (Table 1) (Wild et al. 2010). The total lipid and fatty acid production of the six yeasts was not the same as reported in previous studies (Table S1), for example, the lipid content of *S. cerevisiae*, *L. starkeyi*, and *R. toruloides* have been reported to be 4.3, 55, and 58.3% of DCW (Calvey et al. 2016; Runguphan and Keasling 2014; Wu et al. 2011; Zhao et al. 2010). These differences may be due to use of different strains, different cultivation medium, different biomass harvesting time, and different yeast growth conditions.

Though some yeast species, such as *Y. lipolytica* and *L. starkeyi*, have been used or suggested for CBL production before (Papanikolaou and Aggelis 2011; Papanikolaou et al. 2003; Wu et al. 2011), our analyses showed that they might not be the optimal hosts for CBL production with excess glucose as substrate. *L. starkeyi* is not well suited for CBL production due to its slow growth, and for *Y. lipolytica*, the total lipid production and the potential CBL content in NLM medium was relatively low compared to the other oleaginous yeasts (Table 2 and Fig. 1). Though the lipid content of *Y. lipolytica* can be increased after metabolic engineering, its C16:1 and C18:2 fatty acids contents are usually high, suggesting that *Y. lipolytica* might not be ideal for CBL production (Edem 2002; Jahurul et al. 2013; Lipp and Anklam 1998). *S. cerevisiae* was the only non-oleaginous yeast tested in this study, but its lipid composition was quite different from CBL, suggesting that *S. cerevisiae* is not suitable for CBL production in the near future. Besides, *Y. lipolytica*, *S. cerevisiae*, and *R. graminis* contained relatively low proportions of potential POP, POS, and SOS, further implying that these yeasts would not be the first choice as CBL producers (Fig. 1). Though the potential SOS content of the TAGs extracted from *T. oleaginosus* and *R. toruloides* was low, the two yeasts had a relatively high proportion of potential POP and POS, indicating that they are promising CBL producers (Fig. 1). As *T. oleaginosus* additionally produced higher amounts of TAG than the other yeasts, it is the most promising potential CBL producer among the six yeasts evaluated.

The TAG and fatty acid compositions of *T. oleaginosus* are different from CB (Lipp and Anklam 1998; Zhang et al. 2014). CBL production would require a higher ratio of C18:0 and less polyunsaturated fatty acids (Table 3). Metabolic engineering can assist to alter yeast lipid profiles (Nielsen 2009; Nielsen and Keasling 2016), and further engineering is therefore required to enable production of CBL using *T. oleaginosus*. As genome information of *T. oleaginosus* is available and genetic engineering methods of *T. oleaginosus* to produce modified fatty acids have been successfully established (Görner et al. 2016; Kourist et al. 2015), engineering genes in the lipid biosynthetic pathway, such as genes involved in fatty acid elongation and desaturation, might enable an increased CBL production (Martin et al. 2007; Oh et al. 1997; Toke and Martin 1996). In addition, formation of TAGs is mainly catalyzed by three different enzymes, glycerol-3-phosphate acyltransferase (GPAT), lysophosphatidyl acyltransferase (LPAT), and diacylglycerol acyltransferases (DGAT), which are responsible for adding acyl residues to the *sn*-1, *sn*-2, and *sn*-3 positions of the TAG backbone glycerol, respectively (Bates et al. 2013; Benghezal et al. 2007; Oelkers et al. 2002; Zheng and Zou 2001). As CBL proportion was relatively low, it is important to engineer yeast GPAT, LPAT, and DGAT genes or to express GPAT, LPAT, and DGAT genes specific for CBL production besides altering the C18:0 composition in the total fatty acid pool.

## Electronic supplementary material

ESM 1 (DOCX 1820 kb).
