# Supplementary material for: Cocoa butter-like lipid production ability of non-oleaginous and oleaginous yeasts under nitrogen-limited culture conditions
Source: Appl Microbiol Biotechnol. 2017 Feb 6;101(9):3577–85. doi: 10.1007/s00253-017-8126-7 (PMC5395598; doi:10.1007/s00253-017-8126-7)
Supplement: Supplementary file 1 — (DOCX 1820 kb). [file 253_2017_8126_MOESM1_ESM.docx]

**Supplementary material**

**Cocoa butter****-like lipid production ability of non-oleaginous and oleaginous yeasts under nitrogen limited culture conditions**

**Yongjun Wei^1,2^ ·Verena Siewers^1,2^ · Jens Nielsen^1,2,3^**

**^1^** Department of Biology and Biological Engineering, Chalmers University of Technology, SE-41296 Gothenburg, Sweden

**^2^** Novo Nordisk Foundation Center for Biosustainability, Chalmers University of Technology, SE-41296 Gothenburg, Sweden

**^3^** Novo Nordisk Foundation Center for Biosustainability, Technical University of Denmark, DK-2800 Kgs. Lyngby, Denmark

**Corresponding author**

Jens Nielsen

E-mail: [nielsenj@chalmers.se](mailto:nielsenj@chalmers.se)

Telephone: +46 (0)31 772 3804

Fax: +46(0)31 772 3801

Number of pages: 6 (including cover page)

Number of figures: 2

Number of tables: 3

**
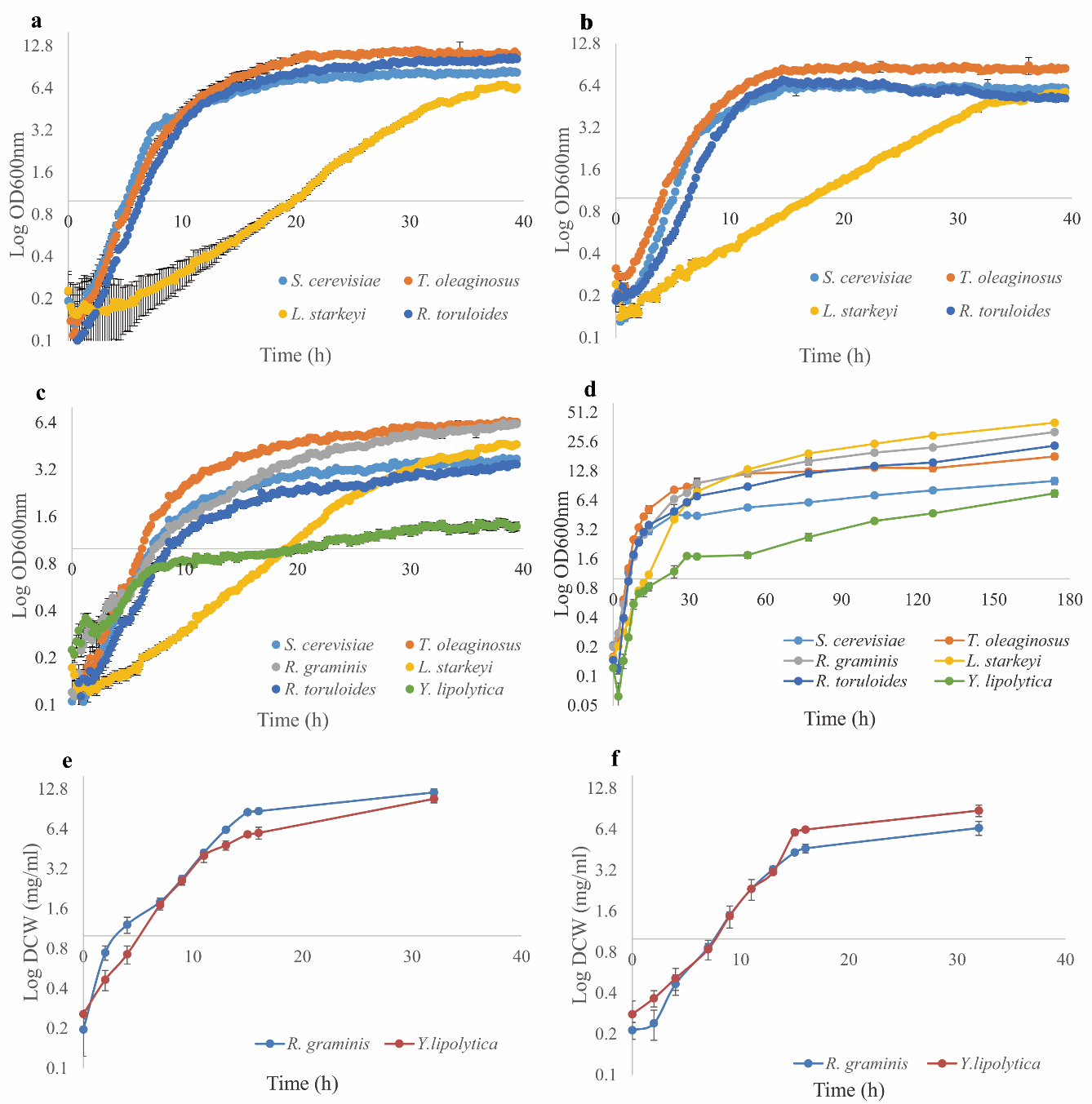
**

**Fig. S1** Growth characteristics of six yeast strains cultivated in different media and conditions. (**a**) Four yeast strains grown in YPD medium in a Bioscreen C (n=8); (**b**) Four yeast strains grown in YM medium in a Bioscreen C (n=8); (**c**) Six yeast strains grown in NLM medium in a Bioscreen C (n=8); (**d**) Six yeast strains grown in 100 ml shake flasks with 20 ml NLM medium (n=3); (**e**) Two yeast strains grown in YPD medium in 500 ml shake flasks with 100 ml YPD medium (n=3) ; (**f**) Two yeast strains grown in YM medium in 500 ml shake flasks with 100 ml YM medium (n=3).


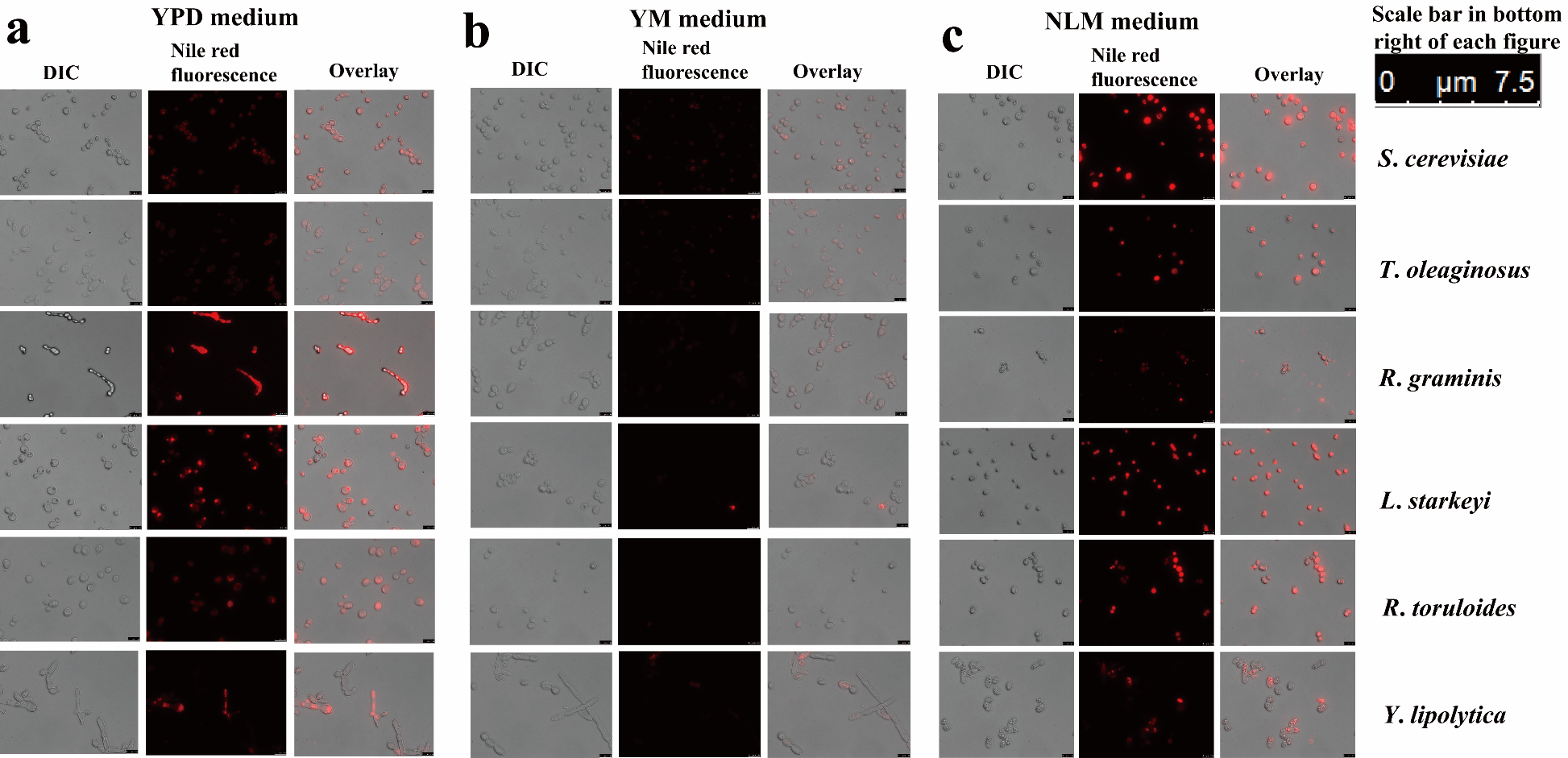


**Fig. S2** Phenotypes and lipid droplets of six different yeasts cultivated in (a) YPD medium; (b) YM medium and (c) NLM medium.

Table S1 Total lipid content including standard deviation of six yeast strains cultivated in NLM medium (n=2)

|  | Fatty acids (mg/g DCW)^a^ | | | | | | | | | |
| --- | --- | --- | --- | --- | --- | --- | --- | --- | --- | --- |
|  | Steryl esters | Triacylglycerol | Ergosterol | Cardiolipin | PE^b^ | PC^b^ | PS^b^ | PI^b^ | Total |  |
| *S. cerevisiae* | 25.9 ± 1.3 | 38.7 ± 5.6 | 4.9 ± 0.6 | ND^c^ | 1.5 ± 0.5 | 4.4 ± 1.1 | 0.9 ± 0.4 | ND | 76.3 ± 9.5 |  |
| *T. oleaginosus* | 0.2 ± 0.1 | 378.6 ± 89.2 | 15.9 ± 0.7 | 0.3 ± 0 | 4.5 ± 0.5 | 14.9 ± 2 | 13.8 ± 1.2 | 0.6 ± 0.7 | 428.8 ± 92.8 |  |
| *R. graminis* | 14.9 ± 8.5 | 183.3 ± 9.9 | 2.6 ± 0.7 | ND | 3.6 ± 0.1 | 8.4 ± 0.2 | ND | ND | 212.9 ± 1 |  |
| *L. starkeyi* | 0.7 ± 0.1 | 164.8 ± 8.7 | 1.4 ± 0.2 | ND | 4 ± 0.1 | 6.3 ± 0 | 3.3 ± 0.6 | 2 ± 0.5 | 182.6 ± 10 |  |
| *R. toruloides* | 2.2 ± 0.8 | 149.4 ± 6.4 | 2 ± 0.1 | ND | 2.6 ± 1 | 7.9 ± 1.7 | 1 ± 0 | 1.3 ± 0.9 | 166.4 ± 10.9 |  |
| *Y. lipolytica* | 5.5 ± 0.1 | 106 ± 8.4 | 3.9 ± 0.4 | 1.8 ± 0.3 | ND | 16.9 ± 0.5 | ND | ND | 134.1 ± 7.1 |  |

^a^ Phosphatidic acid and free fatty acid levels were not determined in this study (below the detection limit or no detectable peaks)

^b^ PE is phosphatidylethanolamine; PC is phosphatidylcholine, PS is phosphatidylserine, and PI is phosphatidylinositol

^c^ ND: not determined (the peak was not detected or the peak area was too small to be determined)

Table S2 Total fatty acid content including standard deviation of six yeast strains cultivated in NLM medium (n=2)

|  | Fatty acids (mg/g DCW) | | | | | | | | | | | | | | | |
| --- | --- | --- | --- | --- | --- | --- | --- | --- | --- | --- | --- | --- | --- | --- | --- | --- |
|  | C12:0 | C14:0 | C14:1 | C16:0 | C16:1 | C18:0 | C18:1 | C18:2 | C18:3 | C20:0 | C20:1 | C22:0 | C24:0 | C26:0 | Total |  |
| *S. cerevisiae* | 0.4 ± 0 | 0.4 ± 0 | 0.5 ± 0.1 | 10.5 ± 0.2 | 38.3 ± 1.1 | 2.7 ± 0.1 | 22.9 ± 0.9 | ND | ND | 0.1 ± 0 | 0.5 ± 0.2 | 0 | 0.1 ± 0 | 0.1 ± 0 | 76.4 ± 1.9 |  |
| *T. oleaginosus* | 0.2 ± 0 | 1.4 ± 0.2 | ND | 73.3 ± 3.5 | 2 ± 0.2 | 15.7 ± 0.1 | 119.1 ± 1.4 | 24.3 ± 1.6 | 0.8 ± 0 | 0.8 ± 0.1 | 3.7 ± 0.5 | 0.3 ± 0 | 4.6 ± 0.8 | 0.3 ± 0.1 | 246.5 ± 2.6 |  |
| *R. graminis* | 0.1 ± 0 | 2.5 ± 0 | ND | 46.3 ± 0.7 | 5.6 ± 1.4 | 3.6 ± 0.1 | 95 ± 0.1 | 38 ± 1.8 | 4 ± 0.1 | 0.6 ± 0 | 3.5 ± 0.7 | 0.5 ± 0 | 3.1 ± 0.1 | 0.2 ± 0 | 202.8 ± 1.5 |  |
| *L. starkeyi* | ND^a^ | 0.6 ± 0.1 | ND | 62.4 ± 0.6 | 3.3 ± 0.1 | 20 ± 0.1 | 94.4 ± 0.9 | 8.3 ± 1.6 | 0.1 ± 0 | 1.4 ± 0 | 3 ± 0.6 | 0.6 ± 0 | 3.1 ± 0 | 0.1 ± 0 | 197.2 ± 0.5 |  |
| *R. toruloides* | 0.2 ± 0.1 | 2.6 ± 0.1 | ND | 51.5 ± 4.1 | 1 ± 0 | 27.3 ± 5.3 | 68 ± 4.5 | 27.5 ± 5.6 | 3.4 ± 0.5 | 0.9 ± 0.1 | 2.3 ± 0.3 | 0.4 ± 0.1 | 1 ± 0.1 | 0 | 186.2 ± 20.7 |  |
| *Y. lipolytica* | ND | 0.2 ± 0 | ND | 22 ± 0 | 39 ± 0.9 | 3.5 ± 0.3 | 74.9 ± 0.7 | 18.2 ± 1.4 | ND | 0.2 ± 0 | 1.8 ± 0.3 | 0.1 ± 0 | 1.9 ± 0 | 0.2 ± 0 | 162 ±0.4 |  |

^a^ ND: not determined (the peak was not detected or the peak area was too small to be determined)

Table S3 The relative TAG content (<5%) including standard deviation of six yeasts cultivated in NLM medium (n=2)

|  | Relative TAG content of TAGs (%) | | | | | |
| --- | --- | --- | --- | --- | --- | --- |
|  | *S. cerevisiae* | *T. oleaginosus* | *R. graminis* | *L. starkeyi* | *R. toruloides* | *Y. lipolytica* |
| TAG (C16:0, C16:1, C16:1) | 2.49 ± 0.13 | 0.08 ± 0.01 | 0.71 ± 0 | 0.04 ± 0 | 1.59 ± 0.01 | 2.32 ± 0.15 |
| TAG (C16:0, C18:3, C18:1) | 0.00 | 0.33 ± 0.01 | 2.8 ± 0.07 | 0.21 ± 0.01 | 0.85 ± 0.01 | 1.28 ± 0.31 |
| TAG (C18:0, C18:2, C18:1) | 0.27 ± 0.10 | 2.04 ± 0.22 | 1.19 ± 0.06 | 0.95 ± 0.07 | 2.59 ± 0.16 | 1.13 ± 0.05 |
| TAG (C22:0, C18:1, C18:1) | 0.04 ± 0.05 | 0.2 ± 0.02 | 0.41 ± 0.02 | 0.56 ± 0 | 0.17 ± 0.01 | 0.79 ± 0.03 |
| TAG (C24:0, C18:1, C18:1) | 0.05 ± 0.07 | 0.79 ± 0.04 | 0.56 ± 0.02 | 0.66 ± 0.06 | 0.08 ± 0.01 | 0.65 ± 0.03 |
| TAG (C20:0, C18:1, C20:0) | 0.06 ± 0.08 | 1.56 ± 0.03 | 0.88 ± 0.06 | 1.61 ± 0.09 | 0.41 ± 0.01 | 0.44 ± 0.12 |
| TAG (C14:0, C18:1, C16:0) | 1.29 ± 0.19 | 0.77 ± 0.2 | 1.67 ± 0.11 | 0.67 ± 0.09 | 2.68 ± 0.03 | 0.42 ± 0.11 |
| TAG (C14:0, C18:2, C16:0) | 0.59 ± 0.19 | 0.08 ± 0.03 | 0.29 ± 0.02 | 0.01 ± 0.02 | 0.24 ± 0.02 | 0.33 ± 0.05 |
| TAG (C20:0, C18:1, C18:1) | 0.1 ± 0.01 | 0.17 ± 0.02 | 0.2 ± 0.01 | 0.62 ± 0 | 0.11 ± 0.01 | 0.28 ± 0.01 |
| TAG (C16:0, C16:0, C14:0) | 0.18 ± 0.25 | 0.07 ± 0.08 | 0.19 ± 0.05 | 0.19 ± 0.07 | 0.18 ± 0.06 | 0.28 ± 0.07 |
| TAG (C16:0, C18:0, C18:0) | 0.00 | 0.31 ± 0.01 | 0.41 ± 0.01 | 0.22 ± 0.03 | 0.14 ± 0.02 | 0.26 ± 0.11 |
| TAG (C20:0, C18:2, C18:1) | 0.08 ± 0.01 | 0.1 ± 0.03 | 0.24 ± 0.03 | 0.24 ± 0.08 | 0.59 ± 0.1 | 0.2 ± 0.11 |
| TAG (C18:0, C18:1, C20:0) | 0.08 ± 0.12 | 0.48 ± 0.01 | 0.61 ± 0.01 | 1.15 ± 0.06 | 0.72 ± 0.04 | 0.12 ± 0.03 |
| TAG (C16:0, C16:0, C18:0) | 0.14 ± 0 | 0.17 ± 0.1 | 0.34 ± 0.04 | 0.37 ± 0.04 | 0.28 ± 0.01 | 0.11 ± 0.01 |
| TAG (C18:0, C18:2, C18:0) | 0.00 | 0.11 ± 0.02 | 0.15 ± 0.11 | 0.13 ± 0.01 | 0.59 ± 0.04 | 0.03 ± 0.05 |
| TAG (C16:0, C18:3, C16:0) | 0.00 | 0.56 ± 0 | 4.04 ± 0.1 | 0.22 ± 0.02 | 2.99 ± 0.04 | 0.00 |
| TAG (C18:0, C18:3, C18:3) | 0.00 | 0.82 ± 0.01 | 2.54 ± 0.12 | 0.19 ± 0.1 | 0.49 ± 0.02 | 0.00 |
| TAG (C18:0, C18:2, C18:2) | 0.00 | 0.79 ± 0.06 | 1.73 ± 0.01 | 1.12 ± 0.01 | 0.32 ± 0.46 | 0.00 |
| TAG (C16:0, C16:0, C16:0) | 0.00 | 0.4 ± 0.56 | 0.08 ± 0.11 | 0.5 ± 0.19 | 0.32 ± 0.11 | 0.00 |
| TAG (C24:0, C18:2, C18:1) | 0.00 | 0.00 | 0.00 | 0.00 | 0.04 ± 0 | 0.00 |
| TAG (C18:0, C18:1, C18:0) | 0.27 ± 0.4 | 1.93 ± 0.02 | 0.35 ± 0.04 | 1.97 ± 0.03 | 2.25 ± 0.06 | 0.1 ± 0.03 |
